# A Bioinspired Fluid-Filled Soft Linear Actuator

**DOI:** 10.1089/soro.2021.0091

**Published:** 2023-06-08

**Authors:** Silvia Filogna, Linda Paternò, Fabrizio Vecchi, Luigi Musco, Veronica Iacovacci, Arianna Menciassi

**Affiliations:** ^1^ The BioRobotics Institute, Scuola Superiore Sant'Anna, Pisa, Italy.; ^2^ Department of Excellence in Robotics and AI, Scuola Superiore Sant'Anna, Pisa, Italy.; ^3^ Research Infrastructures for Marine Biological Resources Department, Stazione Zoologica Anton Dohrn, Napoli, Italy.; ^4^ Integrative Marine Ecology Department, Stazione Zoologica Anton Dohrn, Napoli, Italy.; ^5^ Department of Biological and Environmental Sciences and Technologies, University of Salento, Lecce, Italy.

**Keywords:** bioinspired robotics, fluidic actuators, linear actuator, sipunculid worms, soft robotics

## Abstract

In bioinspired soft robotics, very few studies have focused on fluidic transmissions and there is an urgent need for translating fluidic concepts into realizable fluidic components to be applied in different fields. Nature has often offered an inspiring reference to design new efficient devices. Inspired by the working principle of a marine worm, the sipunculid species *Phascolosoma stephensoni* (Sipunculidae, Annelida), a soft linear fluidic actuator is here presented. The natural hydrostatic skeleton combined with muscle activity enables these organisms to protrude a part of their body to explore the surrounding. Looking at the hydrostatic skeleton and protrusion mechanism of sipunculids, our solution is based on a twofold fluidic component, exploiting the advantages of both pneumatic and hydraulic actuations and providing a novel fluidic transmission mechanism. The inflation of a soft pneumatic chamber is associated with the stretch of an inner hydraulic chamber due to the incompressibility of the liquid. Actuator stretch and forces have been characterized to determine system performance. In addition, an analytical model has been derived to relate the stretch ability to the inlet pressure. Three different sizes of prototypes were tested to evaluate the suitability of the proposed design for miniaturization. The proposed actuator features a strain equal to 40–50% of its initial length—depending on size—and output forces up to 18 N in the largest prototypes. The proposed bioinspired actuator expands the design of fluidic actuators and can pave the way for new approaches in soft robotics with potential application in the medical field.

## Introduction

In recent years, various challenges have been tackled to realize highly performing actuators.^1,2^ This is of major interest both at the industrial and research levels, for different applications including robotics. In this field, when switching from traditional rigid-link robots to soft structures, different strategies should be put in place.^3,4^ Particularly, several soft robots have benefited from a nature-inspired design.^5–11^ Indeed, given the energy efficiency of biological systems, they might provide useful insights to develop novel actuators.^8–10^

Fluidic actuators are most commonly used in bioinspired soft systems due to their high energy density and high power-to-weight ratio.^12–14^ Compared with other actuator classes, fluidic ones are well scalable, thus enabling the development of compact solutions with good performance in terms of stretch and output force.^15,16^ In addition, by implementing fluidic actuation strategies, different types of motion can be obtained, including linear motion.^17,18^ Among systems able to stretch under pressurization, we can consider some pneumatic artificial muscles (PAMs).^19^ For instance, a McKibben-like arrangement has been proposed in pneumatic rubber artificial muscle (PRAM),^20^ ratchet-integrated pneumatic actuator,^21^ and hydro muscles,^22,23^ where an opposite operation occurs compared with McKibben. Indeed, while an elastic material is pressurized, a linear extension motion arises and the radial expansion is limited by outer elements, thus maximizing the stretch in the axial direction.

Furthermore, a similar operation can be found in fiber-reinforced design^24^ where the braided sleeve was used to constrain the deformation in the radial direction of the inflated chambers,^25,26^ improve the stretch performance,^27^ and facilitate the manufacturing.^28^

Typically, fluidic actuators can be supplied with gases or liquids, depending on the system requirements. The first benefit is low gas viscosity, which turns particularly advantageous in miniaturized systems with small supply lines. In addition, pneumatic actuators result overall more lightweight and do not require exhaust lines since air does not need to be collected and can be freely evacuated in the environment. On the contrary, liquid-driven solutions are characterized by low fluid compressibility and by a larger output force/torque with higher controllability.^29^ In this framework, when working with fluidic actuators, driving fluid selection must be based on the target application.

A combined solution was proposed by Xiang *et al.*^30^ It includes a pneumatic circuit controlling a hydraulic McKibben actuator by regulating the liquid in a reservoir directly connected to the artificial muscle. This design has demonstrated an increment of the system stiffness range and, consequently, an improvement of its performances, compared with standard McKibben actuators.

**FIG. 1.**
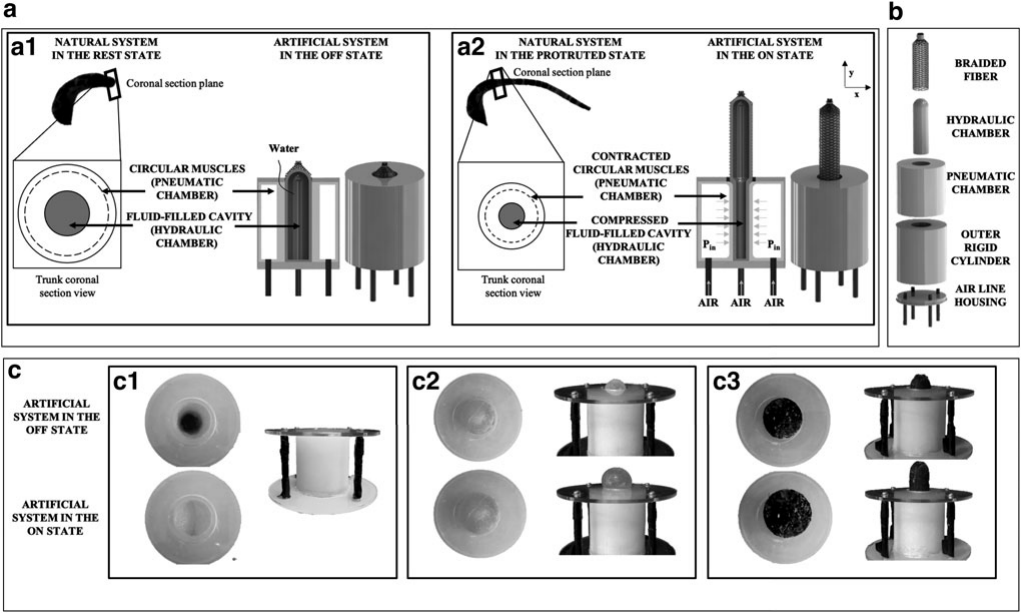
Bioinspired linear actuator concept. **(a)** Illustration of the real organism and schematic representation of the hydrostatic skeleton working principle, before **(a1)** and after **(a2)** activation. In the same panel, the artificial system (in the form of complete module and its section) is also reported, highlighting the correspondence between the natural and artificial components: the natural circular muscles and fluid-filled cavity are mimicked by the artificial pneumatic and hydraulic chamber, respectively. Indeed, the pneumatic chamber causes the squeezing of the fluidic one as the circular muscles and the fluid-filled cavity do. **(b)** Components of the actuator. **(c)** Frontal and lateral view of the actuator components in the rest and actuated state: **(c1)** soft pneumatic actuator within the three-dimensional-printed rigid cylinder; final actuator without **(c2)** and with **(c3)** the fiber braid on the hydraulic part. The upper pictures show the system in rest state, whereas the lower ones show the pressurized system.

Moreover, the combination of gas and liquid allows to explore novel transmission and conversion mechanisms with interesting implications in soft robotics. Indeed, using smart transmission mechanisms for more efficient locomotion or task performance is extremely common in nature.^29,31^ For instance, many invertebrates can produce motion and exert forces thanks to the hydrostatic skeleton that consists of a soft fluid-filled body cavity (coelom) surrounded by muscles. The marine sipunculid worm *Phascolosoma stephensoni* (Sipunculidae, Annelida) is particularly intriguing in this sense. Unlike most annelids, sipunculids are simplified systems with an unsegmented body. Among them, sessile species can evert part of their body by relying on the contraction of circular muscles surrounding the internal fluid-filled cavity. Muscle contraction causes an increase in coelom pressure, resulting in the eversion of a body part rather than in locomotion (Fig. 1a).^32–34^

Taking inspiration from the sipunculid structure and protrusion mechanism, and given the aforementioned advantages provided by combined liquid+gas-based fluidic actuation, a new concept of soft linear fluidic actuator is proposed in this article. Specifically, the actuator contains two soft chambers, a pneumatic one, which acts as the muscular layer, and a fluid-filled internal chamber as in the natural counterpart (Fig. 1a).

Our work aims to push forward the state-of-the-art of soft fluidic systems by developing an innovative linear bioinspired actuator based on an efficient transmission technique. This allows to transmit and convert the pneumatic energy in large output forces in an easy, fast, and safe way.

Moreover, given the increasing demand for small-scale robotic devices, actuator downsizing is an important aspect to take into account.^16^ For this purpose, three different prototype sizes were realized to evaluate the performance at different scales. Detailed design and manufacturing methods are reported. An analytical model was developed and validated to establish the relationship between the inlet pressure and the stretch of the system, depending on the geometric parameters. Finally, stretch and force tests were carried out, and the performance attributes of the new actuator were evaluated and discussed.

## Materials and Methods

### Design and system overview

The new bioinspired actuator consists of two main blocks: a soft pneumatic unit with a hollow cylinder shape, and a water-filled soft chamber, contained therein. The pneumatic block is encased by a rigid cylinder to drive the expansion toward the internal direction (Fig. 1b). When the pneumatic actuator is inflated, it expands radially. As a consequence, the pressure is transferred to the hydraulic chamber that deforms in the axial direction. A fiber braid was placed on the hydraulic chamber to limit the radial expansion and support the linear stretch.

At first, the correct operation of the pneumatic chamber and of the entire actuator was verified. Figure 1c1 reports the expansion of the pneumatic actuator contained in the outer rigid cylinder and without the hydraulic chamber. In the central panel (Fig. 1c2), the stretch of the hydraulic chamber without the fiber braid is illustrated. When the hydraulic chamber is squeezed by the pneumatic actuator, the incompressibility of the internal liquid causes the stretch. However, the expansion occurs both in the axial and radial directions, such as in a balloon. The presence of the fiber braid limits this ballooning effect and maximizes the stretch in the axial direction (Fig. 1c3).

This design combines the advantages of both soft and rigid materials, enabling a compliant and adaptable interface with the environment (i.e., the hydraulic soft chamber) with improved controllability and output force thanks to the rigid case. The rigid case, containing the base of the protrusion mechanism, works as the rocks where the worms stay anchored when protruding the introvert.

Moreover, the design combines the benefits of using both gases and liquids. In particular, the active pneumatic unit enables to reduce the actuator weight and leakages; in addition, the use of air—in light of its low viscosity—favors the miniaturization process. On the contrary, the passive hydraulic component makes it possible to maximize actuator performances (i.e., higher stretches and output forces) and improve both system controllability and setup easiness. Despite this combination (i.e., air outside and liquid inside) being the only one investigated and tested in this article, the proposed structure is flexible enough to switch to a different fluid combination (i.e., air plus air or liquid plus liquid), based on the application requirements.

### Manufacturing

The outer rigid cylinder and the molds for the soft chambers were designed using SolidWorks^®^ and fabricated with UV curable plastic material by three-dimensional (3D) printing (ProJet HD 3000; 3D Systems, South Carolina). The pneumatic and hydraulic chambers were fabricated with silicone by the casting technique. The fiber braid (Pro Power PETBK series, Farnell Components) was thermoformed to the desired diameter.

The assembly starts with the inclusion of the fluidic chamber within the fiber braid (which covers only a part of the chamber and is kept fixed in position by silicone glue) and with the injection of water in the fluidic chamber. The fiber-reinforced hydraulic chamber is placed inside the pneumatic block, coaxial to it. High-friction forces acting at the silicone–silicone interface (namely, where the hydraulic chamber is not covered by the fiber) ensure that the internal chamber does not jump out during the stretch. Last, the two chambers are encased by the outer rigid cylinder.

Once verified the correct operation of the complete actuator (Fig. 1c3), two different silicones were tested to optimize stretch. As a rule of thumb, the silicone used to fabricate the hydraulic chamber was featured by lower (or equal) stiffness compared with that used for the pneumatic one. This choice was motivated by the need for the pneumatic chamber to exert enough force to compress the hydraulic chamber and for the hydraulic one to be sufficiently compliant to promote high stretch.

Three silicone combinations were tested and defined as follows:

1. *Prototype A*: both chambers made of Ecoflex 0030.
2. *Prototype B*: the pneumatic chamber made of Ecoflex 0050 and the hydraulic one of Ecoflex 0030.
3. *Prototype C*: both chambers made of Ecoflex 0050.

A preliminary analysis was performed only on the larger actuator size (*n* = 3 prototypes per material combination) to select the proper silicone combination.

**FIG. 2.**
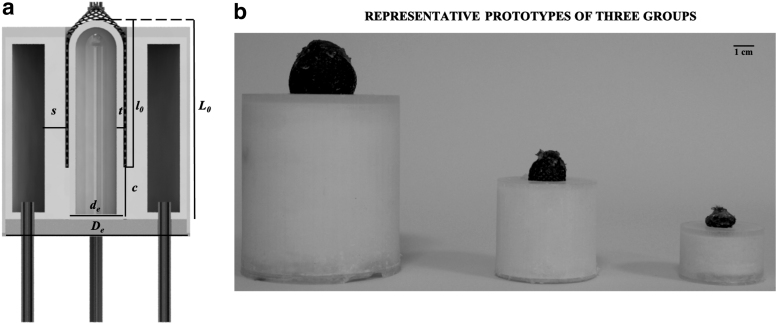
**(a)** Schematic representation of the system geometrical parameters. **(b)** Real picture of one prototype for each group: large, medium, and small. Diameter ($D_{e}$) and internal thickness of the pneumatic chamber (*s*) are shown, as well as the diameter ($d_{e}$), thickness (*t*), and initial length of the hydraulic chamber (*L*_0_), reported as divided into two parts, covered (*l*_0_) and uncovered **(c)** by fiber.

After the material selection, different actuator sizes were analyzed to shed light on the performance variation with dimensions. In analogy with the biological counterpart,^32^ three actuator sizes were identified (large, medium, and small) (Fig. 2 and Table 1) and tested (*n* = 3 prototypes per size). In particular, given the focus on the linear displacement, dimension scaling was applied while halving $d_{e}$ and *L*_0_ when passing from one group to the other. Size scaling enabled to evaluate performance variation with dimension and suitability of the proposed design for miniaturization.

**Table 1.** Geometrical Parameters of the Actuator in the Three Different Scales: Large, Medium, and Small

Groups	$D_{e}$ (mm)	$d_{e}$ (mm)	$L_{0}$ (mm)	$l_{0}$ (mm)	s (mm)	t (mm)	m (g)	V (cm^3^)	V_water_ (mL)
Large	52.0	20.0	60.0	40.0	1.5	6.0	133.07 ± 1.36	127 × 10^3^	12.0
Medium	32.0	10.0	30.0	20.0	1.2	3.0	30.76 ± 0.34	24 × 10^3^	1.0
Small	26.0	5.0	15.0	10.0	1.0	1.5	15.27 ± 0.25	8 × 10^3^	0.1

*D_e_* and *s* identify the diameter and internal thickness of pneumatic chamber, whereas *d_e_* and *t* indicate the same parameters of the hydraulic one. *L*_0_ and *l*_0_ denote the total initial length of the hydraulic chamber and part of the same covered by the fiber, respectively. The remaining length of the hydraulic chamber uncovered by the fiber is indicated by *c* (Fig. 2a). Mass (*m*), actuator volume (*V*), and volume of water filling the hydraulic chamber (*V*_water_) are reported. *m* was experimentally measured. *V* was derived from geometrical parameters. *V*_water_ was geometrically calculated and then experimentally verified.

### Analytical modeling

**FIG. 3.**
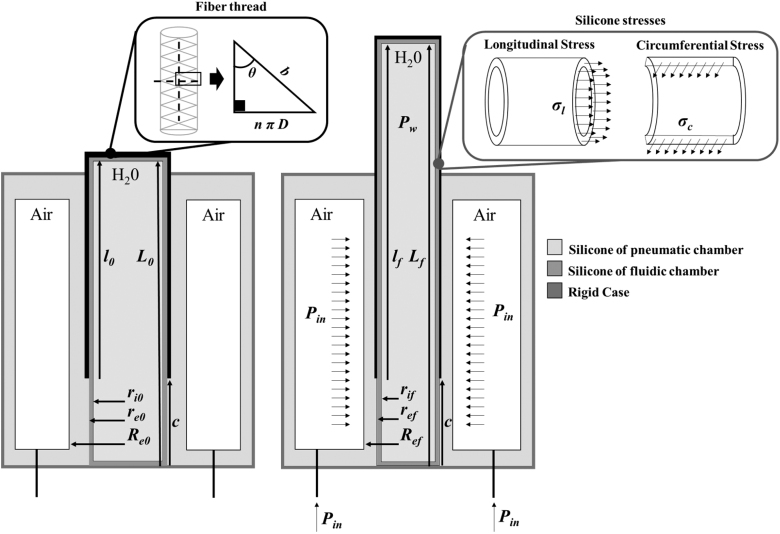
Schematic representation of the bioinspired actuator and physical parameters used in the analytical model. In particular, values indicated by l correspond to the length of the hydraulic chamber portion covered by the fiber thread. These values are different from the length of the entire hydraulic chamber (*L*) as the fiber cover*s* only a part of it (*l*), improving the stretch. The initial and final states of the hydraulic chamber length and radii (inner and outer) are reported as *l*_0_, $l_{f}$, *L*_0_, $L_{f}$, $r_{i0}$, $r_{if}$, $r_{e0}$, $r_{ef}$. Similarly, $R_{e0}$ and $R_{ef}$ are the initial and final radii of the pneumatic cylinder.

A static analytical model has been developed to analyze the relationship between the stretch of the actuator and the inlet pressure of the pneumatic chamber ($P_{in}$) (Fig. 3). Bearing in mind that high-friction forces act at the silicone–silicone interfaces, the model assumes that only the hydraulic chamber portion covered by the fiber elongates, while the uncovered one remains fixed. Therefore, the stretch of the hydraulic chamber, $\lambda_{fiber}$, can be expressed as follows:

(1) $$\lambda_{fiber}=\frac{l_{f}}{l_{0}}=\frac{L_{f}-c}{L_{0}-c}$$

where *l*_0_ and $l_{f}$ are the initial and final lengths of the hydraulic chamber part covered by the fiber, whereas *c* is the constant length of the uncovered portion (Fig. 3). Similarly, *L*_0_ and $L_{f}$ are the initial and final values of the total length of the hydraulic chamber.

The thickness change of the silicone walls during actuation and the related stresses in the longitudinal and circumferential directions have been taken into account during modeling. Indeed, even if these factors are often neglected in fiber-reinforced pneumatic actuators, they result particularly significant in hydraulic soft systems.^35^ The model assumes inextensible fiber, the incompressibility of silicone and water, and a perfect cylindrical shape of the soft chambers before and after pressurization.

Table 2 and Figure 3 report the parameters used in the model. In a steady state, when the output force is zero, the equilibrium equation in the longitudinal direction results^35^:

**Table 2.** Physical Parameters Used in the Analytical Model of the Bioinspired Actuator

Symbol	Description
$F_{fibers}$	Constraint force of the fiber thread
*b*	Thread length
*l*_0_	Length of the hydraulic chamber covered by the fiber thread in the initial state
$l_{f}$	Length of the hydraulic chamber covered by the fiber thread in the final state
*c*	Length of the hydraulic chamber uncovered by the fiber thread
*L*_0_	Total length of the hydraulic chamber in the initial state
$L_{f}$	Total length of the hydraulic chamber in the final state
*n*	Turn number of fiber thread
ϑ	Angle between braided threads
*A_f_*	Cross-sectional area of the silicone wall in the hydraulic chamber
$r_{e0}$	Outer radius of the hydraulic chamber in the initial state
$r_{ef}$	Outer radius of the hydraulic chamber in the final state
$r_{i0}$	Inner radius of the hydraulic chamber in the initial state
$r_{if}$	Inner radius of the hydraulic chamber in the final state
$R_{e0}$	Radius of the inner silicone cylinder of the pneumatic actuator in the initial state
$R_{ef}$	Radius of the inner silicone cylinder of the pneumatic actuator in the final state
$P_{in}$	Inlet pressure
*P_w_*	Hydraulic pressure
$\sigma_{l}$	Silicone longitudinal stress
$\sigma_{c}$	Silicone circumferential stress
$\lambda_{fiber}$	Stretch of the hydraulic chamber covered by the fiber thread

(2) $$P_{w}\pi {r_{if}}^{2}=\sigma_{l}2\pi r_{if}\left(r_{ef}-r_{if}\right)+F_{fibers}$$

To evaluate the constraint force of the fiber braids ($F_{fibers}$), an analytical model typically used to describe McKibben actuators was considered and readapted, as done by Skorina *et al.*^36^ Specifically, it can be expressed as follows:

(3) $$F_{fibers}=P_{w}\left(\frac{b^{2}-3{l_{f}}^{2}}{4\pi n^{2}}-\pi \left({r_{ef}}^{2}-{r_{if}}^{2}\right)\right)$$

Since the hydraulic chamber is modeled as a perfect cylinder, its outer radius, $r_{e}$, and its length, *l*, can be expressed as functions of the angle between fiber braids (ϑ) as follows^37^:

(4) $$r_{e}=\frac{b\sin\vartheta }{2n\pi }$$

(5) l=bcosϑ

To evaluate the longitudinal stress of the silicone material, the Ogden hyperelastic model was used:


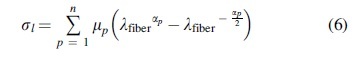

(7) $$\lambda_{fiber}=\frac{l_{f}}{l_{0}}$$

The Ogden model parameters, $\mu_{p}$ and $\alpha_{p}$, were evaluated for n=4 by using experimental stress–strain results of silicone tension tests, performed at an Instron Materials Testing Machine.^38^ By referring to the stress analysis of a cylindrical thin-walled pressure vessel, the circumferential stress, $\sigma_{c}$, was considered twice the longitudinal stress.^39^

According to the volume conservation principle of incompressible materials—as silicone and water are assumed to be—it can be obtained that:

(8) $$r_{if}=\frac{r_{i0}}{\sqrt{\lambda_{fiber}}}$$

(9) $$r_{ef}=\frac{r_{e0}}{\sqrt{\lambda_{fiber}}}$$

(10) $$R_{ef}=\sqrt{{R_{e0}}^{2}+\frac{1-\lambda_{fiber}}{\lambda_{fiber}}{r_{e0}}^{2}}$$

Thus, the inner pressure of the hydraulic chamber, *P_w_*, can be calculated as follows:

(11) $$P_{w}=\frac{\sigma_{l}2\pi r_{if}\left(r_{ef}-r_{if}\right)}{\pi {r_{if}}^{2}+\pi \left({r_{ef}}^{2}-{r_{if}}^{2}\right)-C_{1}}$$

where:

(12) $$C_{1}=\pi {r_{e0}}^{2}\frac{1}{\sin_{}^{2}\vartheta_{0}}\left(1-3{\lambda_{fiber}}^{2}\cos_{}^{2}\vartheta_{0}\right)$$

Finally, the inlet pressure of the pneumatic actuator can be derived from the equilibrium equation in the circumferential direction,^39^ as follows:

(13) $$P_{in}=P_{w}+\frac{\sigma_{c}\left(R_{ef}-r_{if}\right)}{r_{if}}$$

### Performance characterization

**FIG. 4.**
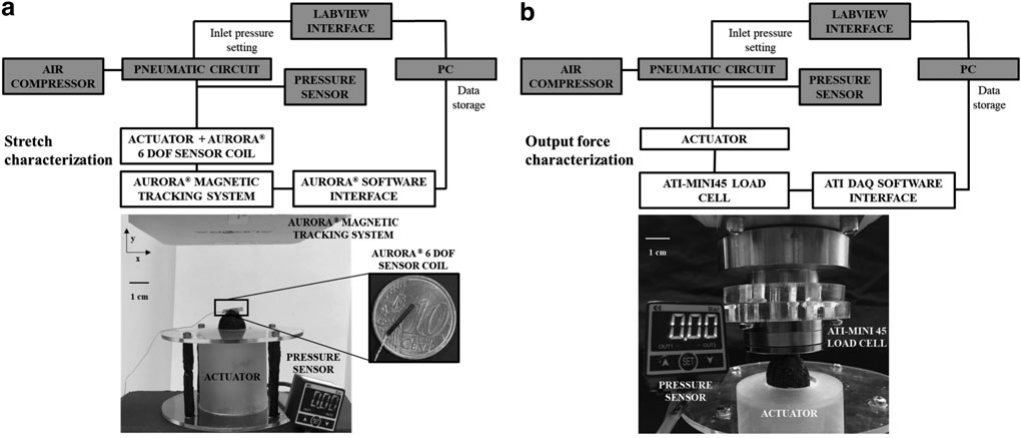
Performance characterization setups. Block diagram and real experimental setup to characterize the actuator in terms of stretch (λ) **(a)** and output force **(b)** are reported. The diagram shows the common (*gray*) and different (*white*) block elements of the two setups. In both of them, a pneumatic circuit connected to an air compressor was used to control the inlet pressure of the actuator. The inlet pressure values were set through a PC by using a LabVIEW interface^40^ and verified by a pressure sensor connected to the system. Based on the type of test performed, the two setups differed in the sensor used: **(a)** for the stretch characterization, the Aurora^®^ 6 DoF sensor coil was fixed on the tip of the actuator to measure its displacement thanks to the magnetic tracking system; and **(b)** for the output force characterization, the ATI-mini45 load cell in contact with the actuator tip was used.

To measure the stretch and output force performances of the actuator, two dedicated experimental setups were used (Fig. 4). The actuator was supplied by a pneumatic system consisting of an air compressor and a pneumatic circuit.^40^ The pressure to be provided to the actuator was set through a LabVIEW interface^40^ and checked by an absolute pressure sensor (SWCN-V01-P3-2; Camozzi Group, Italy). An electromagnetic tracking system (NDI Medical Aurora^®^ Northern Digital, Inc., Waterloo, Canada) and an ATI-mini 45 force/torque sensor (ATI Industrial Automation) were used to evaluate the stretch and output force, respectively (Fig. 4a, b).

Specifically, for the stretch characterization, an Aurora Mini 6 DoF Sensor (0.8 × 9 mm^2^) was fixed on the tip of the fiber-reinforced hydraulic chamber (Fig. 4a) to track and record its displacement while increasing inlet pressure from zero (chamber is at *L*_0_) to the maximum (the chamber reaches $L_{f}$) at a step of 0.2 bar. The maximum pressure value (i.e., $P_{in}$ = 1.4 bar for large and $P_{in}$ = 1.0 bar for medium and small groups) was set according to a trade-off between stretch saturation (no further stretch occurred by increasing pressure) and risk of silicone chambers bursting. Three tests for each pressure value were performed to select the best silicone combination. Likewise, three trials for each pressure value were conducted on three different prototypes for each group (large, medium, and small) to carry out the stretch experiments.

**FIG. 5.**
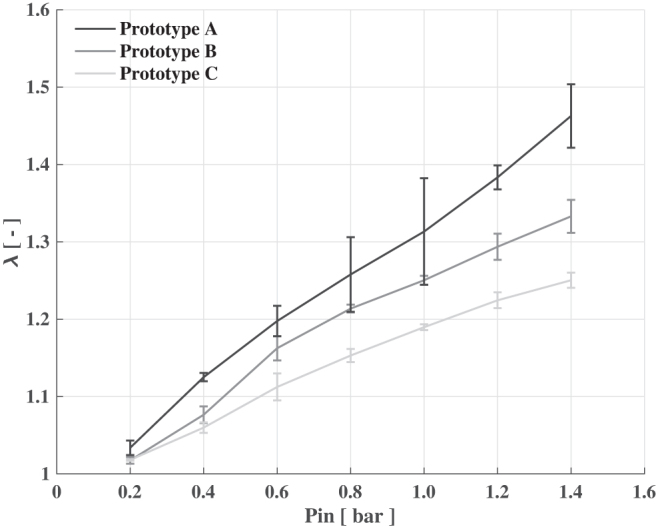
Experimental results of the different prototypes tested. To select the best solution in terms of stretch ($\lambda =L_{f}∕L_{0}$), three types of prototypes were fabricated, by combining diverse silicone materials for the pneumatic and hydraulic chambers: (i) both chambers made of Ecoflex 0030 (Prototype A); (ii) the pneumatic chamber made of Ecoflex 0050 and the hydraulic one of Ecoflex 0030 (Prototype B); and (iii) both chambers made of Ecoflex 0050 (Prototype C). For each class of prototype, three samples were tested with inlet pressures ($P_{in}$) ranging from 0 to 1.4 bar with steps of 0.2 bar. Three tests for each pressure value were performed.

To measure the output forces produced by the actuator, the force sensor was mounted on the end-effector of an industrial robotic arm (Fig. 4b). This easily allowed to fix the force sensor in different positions, corresponding to the stretches of the hydraulic component. Taking into account the maximum stretch of the actuators (Fig. 5) and the need to work in safe ranges to maximize performances while preserving actuator structure, we evaluated the output force when varying λ from 1.00 to 1.30 at steps of 0.05. The pressure range and the step applied for measuring output forces were the same fixed for the stretch test. Three actuators per group were assessed. In addition, we run three tests for each actuator and for each pressure value within groups. This resulted in 72 test runs for the large group and 54 for the medium and small groups. All the data collected from the performance characterization were stored locally.

Moreover, two different abilities of the proposed actuator were demonstrated. First, we proved the capability of each group to lift a mass (*m* = 500, 50 or 5 g based on the group) by providing intermediate (i.e., $P_{in}$ = 0.7 or 0.5 bar) and maximum (i.e., $P_{in}$ = 1.4 or 1.0 bar) input pressure in the actuator's working range. Then, we simulated a standard medical task, namely needle insertion. An actuator from the large group was pressurized ($P_{in}$ = 2.0 bar) to produce linear movement of a needle mounted on the tip of the actuator. A DragonSkin10 (Shore A; Smooth-On, Inc.) silicone sheet of 10 mm was integrated in a human trunk simulator to mimic skin punching and enable *in silico* validation of the proposed scenario.^41^

## Results

### Material selection

Preliminary stretch tests performed on large group samples fabricated with different silicone materials revealed that λ increases almost linearly with the inlet pressure of the pneumatic chamber ($P_{in}$) (Fig. 5). The stretch strongly depends on the compliance of the hydraulic chamber, whereas the influence of the pneumatic chamber stiffness seems not to play a role.

The system entirely made in Ecoflex 0030 (i.e., Prototype A) proved the best solution in terms of stretch, reporting an average maximum value equal to 1.46 ± 0.41 at $P_{in}$ = 1.4 bar. At the same pressure, Prototype B showed a maximum stretch equal to 1.33 ± 0.21, whereas the Prototype C proved a 1.25 ± 0.01 stretch. Despite the latter presenting the lowest stretch compared with the other two, it also has the lower standard deviation values, which implies a higher repeatability (and predictability), thus allowing for an easier control. In this work, we selected the Prototype A as the best solution to obtain maximum stretch but, for applications where a value of 1.25 is sufficient, the Prototype C could be taken into account in light of its greater controllability.

### Performance characterization

**FIG. 6.**
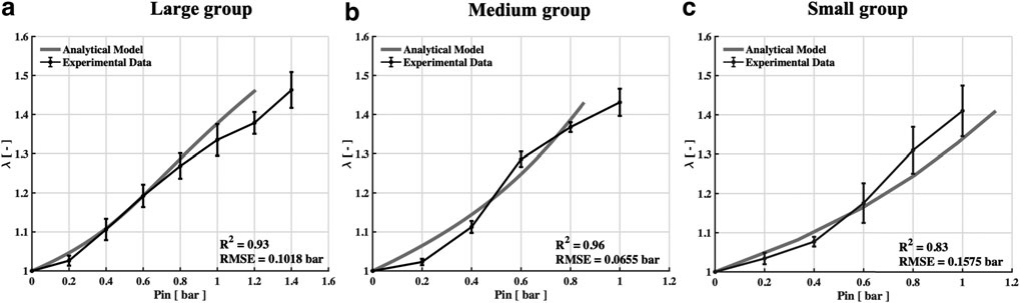
Comparison between the analytical model and experimental results. Stretch of the hydraulic chamber ($\lambda =L_{f}∕L_{0}$ where $L_{f}$ and $L_{0}$ are the final and the initial lengths of the hydraulic chamber, respectively) when varying inlet pressure of the pneumatic chamber ($P_{in}$) for **(a)** large-, **(b)** medium-, and **(c)** small-group actuators. The analytical model is obtained by using Equation (13) that returns the pressure value, given the stretch (as a function of actuator parameters). Experimentally the three groups included three actuators, each tested three times at each pressure value.

Experimental validation was carried out on prototypes with different dimensions (Table 1) to evaluate the system performance and validate the analytical model. In particular, Figure 6 reports a comparison between experimental and analytical model results [Eq. (13)], showing that, at $P_{in}$ = 1 bar, the stretch of the hydraulic chamber was equal to 1.46 ± 0.04, 1.43 ± 0.03, and 1.41 ± 0.06 for the large, medium, and small groups, respectively, thus resulting comparable regardless the downscaling. We can notice that the small group presents greater standard deviations at high-pressure values than other two groups and the trend is less linear. This may depend on several factors related to downsizing: (1) increasing effect of the silicone stresses ($\sigma_{c}$ and $\sigma_{l}$), featured by a hyperelastic behavior [Eq. (6) in the model]; and (2) challenging manufacturing.

A good approximation of the experimental results by the analytical model was proved by evaluating both *R*^2^ and RMSE values (*R*^2^ = 0.93, 0.96, and 0.83 and RMSE = 0.1018, 0.0655, and 0.1575 bar for the large, medium, and small groups, respectively). In particular, for the large and medium groups, the model returns lower pressure values required to achieve a specific stretch, if compared with the experimental results. This is probably due to the friction acting at the silicone–fiber interface, neglected in the analytical model. On the contrary, the lower capability of the model to predict small-group performances may be related to a more significant variance of the experimental data and to the aforementioned downsizing effects. However, taking into account the actuator complexity, these results prove the analytical model goodness to describe the proposed actuation concept behavior.

**FIG. 7.**
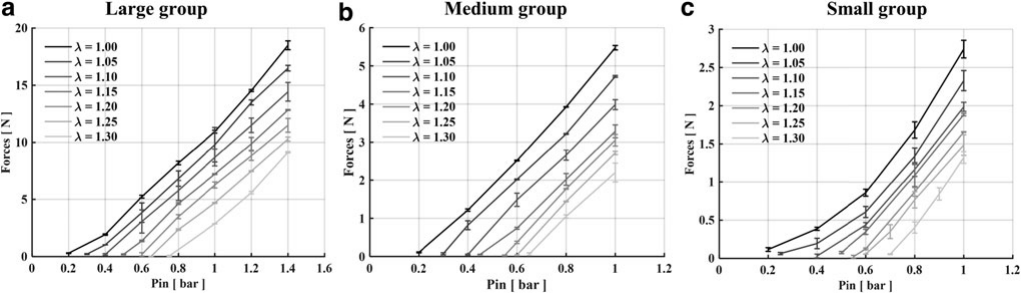
Output forces of **(a)** large, **(b)** medium, and **(c)** small groups, evaluated at seven stretch (λ) values, from 1 to 1.3 at a step of 0.05. Each test was carried out three times on three different actuator prototypes.

The output force at the actuator tip was evaluated for the three size groups when varying input pressure and stretch condition (λ in the range of 1.00:0.05:1.30) (Fig. 7). When considering the large and medium groups, output force increases linearly with pressure at all the considered stretches. Regarding the first group, we can notice a linear trend for each λ to reach maximum output forces from 9.11 ± 0.06 N (λ = 1.30) to 18.50 ± 0.38 N (λ = 1.00) when input pressure reaches 1.4 bar. Medium-size prototypes also follow a linear increase until producing maximum forces from 2.20 ± 0.24 N (λ = 1.30) to 5.47 ± 0.05 N (λ = 1.00) at $P_{in}=1.0$ bar.

Dimension scaling implies a jump into a nonlinear force increase with pressure. This behavior is visible in the large deformation regimen of small actuators where a nonlinear force increase occurs and it enables the small actuators to accomplish maximum output forces in the range 1.32 ± 0.07 N (λ = 1.30) to 2.74 ± 0.11 N (λ = 1.00).

**FIG. 8.**
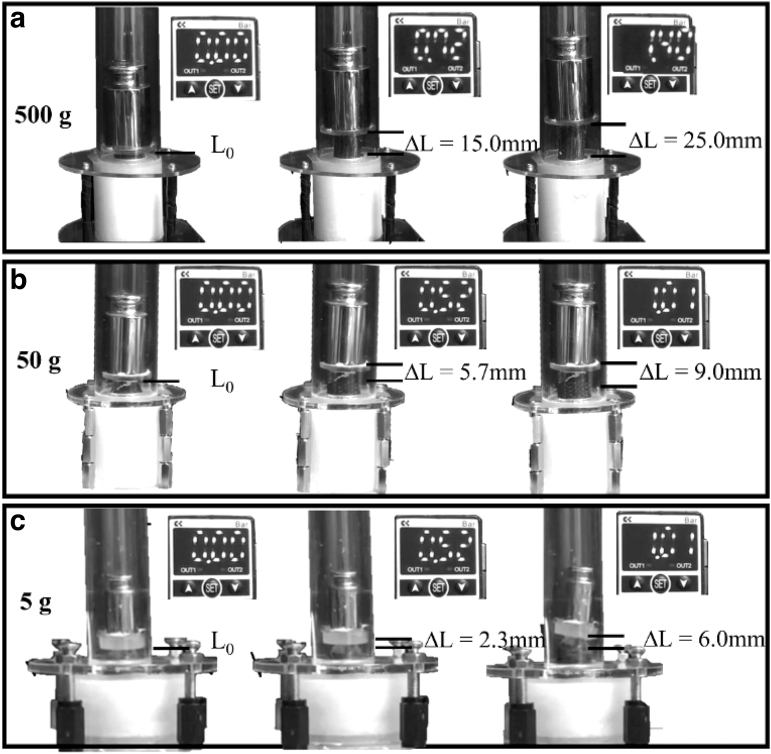
Experimental demonstrative test. All the systems developed were able to stretch and exert forces at the same time by lifting a mass of 500, 50, and 5 g for large **(a)**, medium **(b)**, and small **(c)** groups, respectively. Depending on the dimensions, the pressure values span from 0 bar to 1.4 bar for large samples and from 0 bar to 1.0 bar for medium and small ones. The actuators were able to elongate 15, 6, and 2.5 mm at intermediate pressure values (i.e., $P_{in}$ = 0.7 bar or 0.5 bar based on the group) and 25, 9, and 6 mm at maximum inlet pressures (i.e., $P_{in}$ = 1.4 bar or 1 bar based on the group), respectively.

**FIG. 9.**
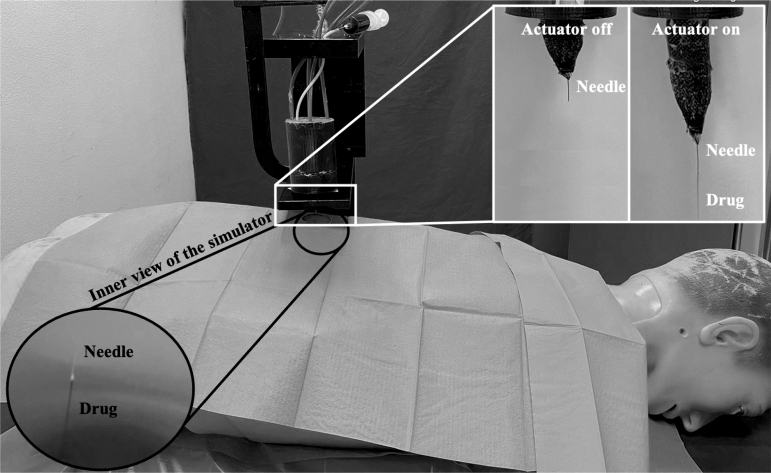
*In silico* needle placement task through the proposed soft linear actuator. When switched on, the actuator protrusion enables needle displacement and skin-like material punching. The possibility to locally inject a drug through the needle is shown. Release during injection outside and inside the phantom of a human simulator is shown.

To assess the capability of the actuators to operate in potential application scenarios, we tested their capability to exert a lift force (Fig. 8) and to perform a standard medical task (Fig. 9). In the first case, the ability of the system to stretch and exert forces at the same time was assessed. In particular, the actuator was able to move for about 25, 9, and 6 mm—at $P_{in}$ = 1.4 (large group) and $P_{in}$ = 1 bar (medium and small groups)—by lifting a mass of 500, 50, and 5g, thus producing forces of about 5, 0.5, and 0.05 N, respectively.

In the second case, we evaluated *in silico* the capability of the actuator to perform needle-based interventions as in minimally invasive surgery. Indeed, given the (1) capability to perform linear motion, (2) the relatively high output force compared with other soft tools, (3) the intrinsic biocompatibility associated with the fully polymeric (silicone and printable plastic materials) structure, and (4) the safe interaction with the host associated with the fluidic chamber performing as an active unit, this actuator design might be advantageously used for a set of medical tasks.

We therefore integrated a needle on the actuator tip and exploited its linear motion to let the needle reach a target location. The exerted force was sufficient to punch a 10 mm DragonSkin10 skin-like silicone layer, thus proving the capability of the actuator to operate in the proposed application either for biopsy or for targeted drug injection (see also Supplementary Video S1). Given the metal-free actuator structure, it might be eligible in the future as a component for a magnetic resonance imaging (MRI)-compatible needle placement soft robot.

## Discussion and Conclusion

Soft linear actuators are typically inflatable systems in which the stretch is obtained by direct pressurization. The main goal of this work was to develop a new soft efficient actuator where the linear motion is enabled by a bioinspired fluidic transmission mechanism.

In this work, after selecting the best silicone combination, experimental results demonstrated that all three groups achieved strains between 40% and 50% at 1 bar, regardless of dimensions. The relationship between stretch and inlet pressure was well predicted by the proposed analytical model that fitted with the experimental results, showing an RMSE value equal to about 0.11, 0.07, and 0.16 bar, for large, medium, and small groups, respectively. Although it can be considered a good fitting, the model underestimated the required inlet pressures for the large and medium groups due to neglected friction forces, and showed lower capability to predict the behavior of the small group due to downscaling effects. In addition, a more linear trend was verified in the large-group samples than the other two groups. However, we should notice that, at the same input pressure, the stretch percentage was similar for all three groups.

All these findings are interesting because the elongating component (i.e., hydraulic chamber) is not directly actuated, but it is a passive chamber that deforms due to a transmission system. Large-group prototypes were able to stretch up to 27 mm proving to be competitive with state-of-the-art systems. For instance, among the PAM actuators, a reverse PAM^36^ with 50 mm total length and 11.54 mm nominal diameter was able to elongate about 23 mm at 1.5 bar. Similarly, the PRAM^20^ actuator with 80 mm in length and 8.4 mm diameter was able to expand the length just for 10 mm under a pressure of 1.6 bar. Moreover, Zhang *et al.*^42^ explored the design of a fiber-reinforced flexible pneumatic actuator able to bend and elongate. Prototypes with a length of 65 and 15 mm diameter, reached stretches of 14 mm at 1.3 bar.

In parallel, the authors are aware that in the wide plethora of soft fluidic actuators, there are many other direct pressurized systems capable of better performances than the proposed actuator. For instance, single modules of STIFFness controllable Flexible and Learnable manipulator for surgical OPerations (STIFF-FLOP)-like manipulators can achieve maximum elongation of 64%^26^ to 100%^28^ depending on the design. Likewise, some McKibben-like muscles can accomplish elongation of two^43^ or three-and-half times^21^ their initial length.

Output force was evaluated by using a specific experimental setup, including a force sensor. Maximum forces were about 19, 5.5, and 3 N for large, medium, and small groups, revealing the downscaling impact on force. Similar to stretch, a more linear trend occurred for the large group with respect to the medium and small ones. This could be ascribed to the fact that the inner chamber in the smaller prototypes was completely squeezed by the pneumatic chamber at higher inlet pressures. Therefore, all the water was pushed toward the tip, increasing the exerted force. As concerns force, our solution has shown better or comparable performance with respect to traditional soft pneumatic actuators.

The aforementioned systems have also been characterized in terms of output force. For instance, the fiber-reinforced soft actuator proposed by Zhang *et al.*^42^ showed an output force of about 4.5 N at 0.7 bar compared with 8 N of our actuator of similar dimensions at the same inlet pressure. On the contrary, the PRAM actuator^20^ reported a force value of about 2.5 N at $P_{in}$ = 1.5 bar. Lastly, we can state that our system achieved output force values of about 20 N comparable with those of the mentioned manipulators' single module.^26,28^

Overall, the performance of an actuator depends on many factors, such as geometrical parameters, mass, volume, strain, and forces, from which it is possible to derive the actuator performance metrics.^44,45^ Table 3 reports a list of indices calculated on the basis of the parameters discussed so far. Referring to the performance metrics of traditional pneumatic and hydraulic systems,^45^ we can define how the two components act in our system. A considerable transducer efficiency, between 30% and 60% was verified, depending on the size and the stretch value considered. Noticeable small samples proved high efficiency, underlining the downsizing potential on the performances. Furthermore, high efficiency is also due to the liquid incompressibility and no heating losses, which are the peculiarities of hydraulic transmission.

**Table 3.** Starting from Stretch and Forces Experimental Results, a Number of Performance Metrics Were Calculated for the Proposed Actuator

Group	Large	Medium	Small
Density (g/cm^3^)	1.04	1.27	1.91
Maximum stretch (mm)	27.00	13.50	6.75
Maximum strain (%)	46.28 ± 4.59	43.10 ± 3.48	41.01 ± 6.46
Maximum output force (N)			
λ = 1.00	18.50 ± 0.38	5.47 ± 0.05	2.74 ± 0.11
λ = 1.05	16.49 ± 0.24	4.72 ± 0.02	2.33 ± 0.13
λ = 1.10	14.43 ± 0.81	3.98 ± 0.13	1.98 ± 0.06
λ = 1.15	12.84 ± 0.03	3.28 ± 0.17	1.88 ± 0.01
λ = 1.20	11.50 ± 0.60	3.05 ± 0.15	1.65 ± 0.004
λ = 1.25	10.29 ± 0.18	2.71 ± 0.04	1.48 ± 0.13
λ = 1.30	9.11 ± 0.06	2.20 ± 0.24	1.32 ± 0.07
Maximum stress (Pa)			
λ = 1.00	29.4	34.8	69.8
λ = 1.05	26.2	30.0	59.3
λ = 1.10	23.0	25.3	50.4
λ = 1.15	20.4	20.9	47.9
λ = 1.20	18.3	19.4	42.0
λ = 1.25	16.4	17.3	37.7
λ = 1.30	14.5	14.0	33.6
Work capacity (J)			
λ = 1.00	—	—	—
λ = 1.05	49.47 × 10^−3^	7.10 × 10^−3^	1.75 × 10^−3^
λ = 1.10	86.58 × 10^−3^	11.94 × 10^−3^	29.7 × 10^−3^
λ = 1.15	115.60 × 10^−3^	14.76 × 10^−3^	4.23 × 10^−3^
λ = 1.20	138.00 × 10^−3^	18.30 × 10^−3^	4.95 × 10^−3^
λ = 1.25	154.35 × 10^−3^	20.32 × 10^−3^	5.55 × 10^−3^
λ = 1.30	163.98 × 10^−3^	19.80 × 10^−3^	5.94 × 10^−3^
Specific energy (J/kg)			
λ = 1.00	—	—	—
λ = 1.05	0.37	0.23	0.11
λ = 1.10	0.65	0.39	0.19
λ = 1.15	0.87	0.48	0.27
λ = 1.20	1.03	0.59	0.32
λ = 1.25	1.16	0.66	0.36
λ = 1.30	1.23	0.64	0.38
Energy density (J/m^3^)			
λ = 1.00	—	—	—
λ = 1.05	388.23	293.44	219.42
λ = 1.10	679.47	494.87	372.93
λ = 1.15	906.90	611.75	531.14
λ = 1.20	1083.00	758.47	621.55
λ = 1.25	1211.30	842.40	696.89
λ = 1.30	1286.90	820.64	745.86
Efficiency (−)			
λ = 1.00	—	—	—
λ = 1.05	0.39	0.63	1.24
λ = 1.10	0.36	0.55	1.11
λ = 1.15	0.33	0.48	1.11
λ = 1.20	0.31	0.46	1.00
λ = 1.25	0.29	0.43	0.94
λ = 1.30	0.26	0.36	0.87
Power-to-weight ratio (W/kg)			
λ = 1.00	—	—	—
λ = 1.05	0.12	0.11	0.11
λ = 1.10	0.22	0.19	0.19
λ = 1.15	0.29	0.24	0.27
λ = 1.20	0.34	0.29	0.32
λ = 1.25	0.38	0.33	0.36
λ = 1.30	0.41	0.32	0.38
Power density (W/m^3^)			
λ = 1.00	—	—	—
λ = 1.05	0.972 × 10^3^	4.769 × 10^3^	14.369 × 10^3^
λ = 1.10	1.702 × 10^3^	8.044 × 10^3^	24.442 × 10^3^
λ = 1.15	2.271 × 10^3^	9.943 × 10^3^	34.783 × 10^3^
λ = 1.20	2.712 × 10^3^	12.329 × 10^3^	40.704 × 10^3^
λ = 1.25	3.034 × 10^3^	13.693 × 10^3^	45.637 × 10^3^
λ = 1.30	3.223 × 10^3^	13.339 × 10^3^	48.845 × 10^3^
Work to lift mass (J) (λ max)	122.6 × 10^−3^	4.4 × 10^−3^	0.29 × 10^−3^

In detail, maximum output force values in seven different λ values (from λ = 1.00 to λ = 1.30 by step of 0.05) were used to determine the maximum stress. Then, the work performed to lift a specific mass (*m* = 500, 50, and 5 g for large, medium, and small groups, respectively) was calculated for each group. Specific work, work density, efficiency, power-to-weight ratio, and power density were derived by using the work and geometrical parameters.

With regard to the power-to-weight ratio, we obtained a value spanning from 0.10 to 0.40 W/kg regardless of sample sizes, but depending only on the considered λ. Although the weight of the prototypes differs for one or two orders of magnitude between the groups, the ratio remains constant indicating that it follows the dimension scaling down and depends only on power.

To compare our actuator with different actuation strategies, we refer to the graphical method proposed by Zupan *et al.*^44^ By analyzing each graph, we could confirm that our system performance metrics are slightly lower than those of pneumatic systems. This is a clear indication that, despite part of the pneumatic energy being provided is used to activate the transmission mechanism, the proposed actuator is a competitive fluidic system featured by high efficiency. Nevertheless, it is worth pointing out that the fluidic transmission mechanism built-into the proposed actuator provides a good trade-off between benefits and performance. Indeed, our system is featured by properties highly desired in the robotic field, such as human-safe interaction (particularly relevant in medical applications) and readiness to miniaturization process, at the expense of a slightly lower performance, but remaining competitive with some existing actuators.

In the end, the proposed actuator has the potential to be applied in a range of uses.^46,47^ Among those, its potential in relevant medical tasks such as needle insertion was demonstrated. The MRI-compatibility, intrinsic safety, and readiness for downsizing of the actuator make it suitable for integration in a medical robotic platform and deserve further investigation. Furthermore, the actuator might serve as a valid tool for robots able to adapt to the environment, paving the way for new approaches in soft robotics and enriching the fluidic actuator scenario.

## Supplementary Material

Supplemental data
